# The effect of surgery started at different time point during the day on the clinical outcomes of mitral valve surgery

**DOI:** 10.3389/fcvm.2024.1360763

**Published:** 2024-02-16

**Authors:** Shuai Zheng, Jiangang Wang, Haibo Zhang, Shengyu Wang, Xu Meng

**Affiliations:** Department of Cardiac Surgery, Beijing Anzhen Hospital, Capital Medical University, Beijing, China

**Keywords:** mitral valve surgery, circadian rhythm, cardiopulmonary bypass, clinical outcome, long-term

## Abstract

**Background:**

The clinical prognosis of mitral valve surgery at morning, afternoon, and evening is not yet clear. The aim of the study is to investigate the impact of different time periods of surgery in the morning, afternoon and evening on the short-term and long-term results of mitral valve surgery.

**Methods:**

From January 2018 to December 2020, 947 patients with mitral valve surgery in our department were selected. These patients were divided into 3 groups according to the starting time of surgery. Morning group (operation start time 8:00–10:30, *n* = 231), afternoon group (operation start time 12:00–14:30, *n* = 543), and evening group (operation start time 17:30–20:00, *n* = 173). The short-term and long-term results of the three groups were compared.

**Results:**

There were no significant difference in the long-term mortality, long-term risk of stroke and reoperation. And there were no significant difference in in-hospital outcomes, including mortality, stroke, cardiopulmonary bypass time, aortic cross clamp time, mitral valve repair convert to mitral valve replacement, number of aortic cross clamp ≥2 times, unplanned secondary surgery during hospitalization (including thoracotomy hemostasis, thoracotomy exploration, redo mitral valve surgery, and debridement), intra-aortic balloon pump, extracorporeal membrane oxygenation, continuous renal replacement therapy, mechanical ventilation time, and intensive care unit length of stay.

**Conclusion:**

There is no significant difference in the risk of short-term and long-term survival and adverse events after mitral valve surgery at different time periods in the morning, afternoon, and evening. Mitral valve surgery at night is safe.

## Introduction

In recent years, the total number of cardiovascular surgery in China has shown an overall upward trend. According to the data from Chinese Society of Extra-Corporeal Circulation (1), 725 hospitals in China have carried out over 270,000 cases of cardiovascular surgery in 2021. Among them, there are 30 hospitals with a surgical volume exceeding 2,000 cases, 5 hospitals with a surgical volume exceeding 5,000 cases, Anzhen Hospital and Fuwai Hospital have a surgical volume exceeding 14,000 cases (1). In hospitals with a large number of surgeries, cardiac surgeons not only perform selective surgery in the morning and afternoon, but they also frequently perform selective surgery in the evening. Patient families often doubt about the safety of afternoon surgery and evening surgery, and hope their surgeries can be scheduled in the morning. Is there a difference in the safety of surgery among morning, afternoon, and evening? Cardiac surgeons often provide empirical answers, but cannot provide reliable medical evidence. Recently, several studies have compared the differences in myocardial injury and clinical prognosis between morning and afternoon cardiac surgery, but there are significant differences between research conclusions (2–5). This study retrospectively analyzed the clinical data of patients with mitral valve surgery, aiming to compare the impact of surgery at different time periods in the morning, afternoon, and evening on the perioperative risk and long-term outcomes of mitral valve surgery.

## Methods

### General information

From January 2018 to December 2020, our department conducted 1,016 cases of mitral valve surgery, including single mitral valve surgery, mitral valve surgery combined with tricuspid valve surgery, mitral valve surgery combined Maze IV procedure for atrial fibrillation, mitral valve surgery combined with tricuspid valve surgery and Maze IV procedure. According to the start time of the surgery, patients were divided into three groups: the morning group (surgery start time 8:00–10:30, *n* = 231), the afternoon group (surgery start time 12:00–14:30, *n* = 543), and the evening group (surgery start time 17:30–20:00, *n* = 173). Among 1,016 patients, the start time of the surgery in 69 patients outside the three time periods mentioned above, and ultimately 947 patients were included in this study. We retrospectively collected clinical data of patients who had elective surgery during the study period that meet the inclusion criteria. Among them, there are 501 female patients. Patients aged 12–82 years old, with a body mass index of 12.1–36.6 kg/m^2^. There was no statistically significant difference in baseline data among the three groups (all *P* > 0.05, Table 1). The study was approved by the Ethics Committee of Beijing Anzhen Hospital Affiliated to Capital Medical University (Ethics No. 2020150X). All patients underwent elective surgery and signed informed consent before surgery.

**Table 1:** Patient characteristics.

	Moring group (*n* = 231)	Afternoon group (*n* = 543)	Evening group (*n* = 173)	*P* value
Female, *n*(%)	119 (51.5)	291 (53.6)	91 (52.6)	0.866
Age, y	57.0 (48.0, 64.0)	57.0 (49.0, 63.0)	57.0 (48.0, 64.0)	0.964
BMI, kg/m^2^	24.23 ± 3.11	24.15 ± 3.32	23.86 ± 3.85	0.520
LVEF, %	62.0 (57.0, 66.0)	62.0 (57.0, 66.0)	62.0 (56.5, 66.0)	0.874
LVEDD, mm	51.0 (47.0, 57.0)	50.0 (46.0, 57.0)	52.0 (46.5, 56.0)	0.845
LA, mm	46.0 (41.0, 52.0)	46.0 (42.0, 52.0)	47.0 (41.0, 52.0)	0.465
Hyperlipidemia, *n*(%)	13 (5.6)	28 (5.2)	11 (6.4)	0.829
Diabetes, *n*(%)	14 (6.1)	25 (4.6)	12 (6.9)	0.433
Hypertension, *n*(%)	63 (27.3)	196 (36.1)	60 (34.7)	0.057
COPD, *n*(%)	0 (0.0)	2 (0.4)	0 (0.0)	0.328
Pulmonary hypertension, *n*(%)	6 (2.6)	30 (5.5)	12 (6.9)	0.110
History of cerebral infarction, *n*(%)	3 (1.3)	7 (1.3)	3 (1.7)	0.908
Coronary artery disease, *n*(%)	5 (2.2)	15 (2.8)	5 (2.9)	0.866
Atrial fibrillation, *n*(%)	110 (47.6)	287 (52.9)	85 (49.1)	0.360
Left atrial thrombus, *n*(%)	7 (3.0)	27 (5.0)	6 (3.5)	0.405
Previous cardiac surgery, *n*(%)	8 (3.5)	20 (3.7)	12 (6.9)	0.144
Rheumatic heart disease, *n*(%)	86 (37.2)	217 (40.0)	63 (36.4)	0.620
Regenerative heart disease, *n*(%)	110 (47.6)	253 (46.6)	79 (45.7)	0.925
Congenital heart disease, *n*(%)	5 (2.2)	14 (2.6)	7 (4.0)	0.486
Infectious endocarditis, *n*(%)	7 (3.0)	18 (3.3)	10 (5.8)	0.270
Secondary mitral valve diseases, *n*(%)	20 (8.7)	45 (8.3)	16 (9.2)	0.923
MS, *n*(%)	11 (4.8)	20 (3.7)	4 (2.3)	0.434
MR, *n*(%)	147 (63.6)	323 (59.5)	104 (60.1)	0.551
MS + MR, *n*(%)	73 (31.6)	200 (36.8)	65 (37.6)	0.323
MVr, *n*(%)	30 (13.0)	65 (12.0)	21 (12.1)	0.924
MVr + Maze, *n*(%)	4 (1.7)	5 (0.9)	2 (1.2)	0.649
MVr + TVr, *n*(%)	70 (30.3)	149 (27.4)	52 (30.1)	0.649
MVr + TVr + Maze, *n*(%)	82 (35.5)	213 (39.2)	67 (38.7)	0.614
MVr + TVr + Maze + LAT, *n*(%)	6 (2.6)	20 (3.7)	5 (2.9)	0.704
MVR, *n*(%)	10 (4.3)	8 (1.5)	6 (3.5)	0.051
MVR + Maze, *n*(%)	0 (0.0)	3 (0.6)	0 (0.0)	0.188
MVR + TVr, *n*(%)	11 (4.8)	33 (6.1)	9 (5.2)	0.743
MVR + TVr + Maze, *n*(%)	17 (7.4)	40 (7.4)	10 (5.8)	0.764
MVR + TVr + Maze + LAT, *n*(%)	1 (0.4)	7 (1.3)	1 (0.6)	0.424
Mechanical valve, *n*(%)	18 (7.7%)	37 (6.8%)	10 (5.7%)	0.729
Bioprosthetic valve, *n*(%)	21(9.1%)	54(9.9%)	16(9.2%)	0.920
Minimally invasive surgery, *n*(%)	4(1.7)	8(1.5)	2(1.2)	0.891

COPD, chronic obstructive pulmonary disease; LAT, left atrial thrombus; Maze, maze procedure; MR, mitral regurgitation; MS, mitral stenosis; MVr, mitral valve repair; MVR, mitral valve replacement; TVr, tricuspid valve repair.

### Surgical strategy

All patients underwent mitral valve surgery under general anesthesia, tracheal intubation and hypothermic cardiopulmonary bypass. Nine hundred and thirty three patients received conventional sternotomy, other 14 patients received minimally invasive surgery with 6 cases of incision in the lower sternum and 8 cases of right thoracotomy. In minimally invasive surgery, there are 3 cases of mitral valve repair or replacement combined tricuspid surgery, 11cases of single mitral valve repair or replacement. All patients underwent ascending aorta, inferior vena cava and superior vena cava catheterization to establish extracorporeal circulation. A left heart drainage tube was placed through the right superior pulmonary vein. After perfusing cold cardioplegic at the aortic root and cardiac arrest, mitral valve were explored through the atrial sulcus incision or the right atrium and atrial septum incision. For mitral valve replacement, the diseased mitral valve leaflets are removed, retaining or not retaining the posterior leaflet. The artificial valve prostheses were sutured continuously with 2-0 prolene thread or intermittently with 2-0 polyester thread. All patients with mitral valve repair underwent ring annuloplasty with an artificial ring. In addition to ring reconstruction, the repair techniques used for nonrheumatic mitral valve lesions also include wedge or triangular resection of the posterior leaflet, posterior leaflet folding, artificial chordae implantation, leaflet sliding technique, leaflet widening, and edge to edge technique. Rheumatic mitral valve disease were repaired using our “Score procedure” (6–8): (1) remove the fibrous plaques at the junction; (2) check the natural state of the valve; (3) commissuroplasty; (4) Release of subvalvular apparatus (papillary muscle separation). Patients with tricuspid valve repair were all implanted with tricuspid valve artificial rings. For patients with underdeveloped valve leaflets, autologous pericardial widening was used. Radiofrequency ablation of atrial fibrillation was performed using an modified Maze procedure, which involved ablation of bilateral pulmonary veins, left atrial posterior wall, superior vena cava, inferior vena cava, tricuspid isthmus, mitral isthmus, left atrial appendage, and right atrial appendage. Routine oral administration of warfarin for anticoagulation after surgery, maintaining an INR of 1.8–2.5.

### Endpoints

The primary endpoint was long-term all-cause mortality. The secondary endpoints were stroke, mitral valve reoperation and in-hospital outcomes, including in-hospital mortality, stroke, cardiopulmonary bypass time, aortic cross clamp time, mitral valve repair convert to mitral valve replacement, number of aortic cross clamp ≥2 times, unplanned secondary surgery during hospitalization (including re-thoracotomy for bleeding, re-thoracotomy for pericardial tamponade, redo mitral valve surgery, and debridement), intra-aortic balloon pump (IABP), extracorporeal membrane oxygenation (ECMO), continuous renal replacement therapy (CRRT), mechanical ventilation time, and intensive care unit length of stay.

### Statistical analysis

Continuous variable were expressed as mean ± standard deviation. Categorical variable were given as frequencies and percentages. Student *t* test or nonparametric analysis were used to compare continuous variables. The chi-square test was used to assess categorical variables. A survival analysis was performed using the Kaplan–Meier method. All *p* values were 2-sided, *p* < 0.05 was considered statistically significant. All the statistical analysis were performed using IBM SPSS statistics 26 software (SPSS, Inc., Chicago, IL, USA).

## Result

### Patients characteristics

Among the 947 patients in three groups, 791 cases (83.5%) underwent mitral valve repair, 156 cases underwent mitral valve replacement, and a total of 65 mechanical valves and 91 bioprosthetic valves were implanted. There were 366 cases of rheumatic mitral valve disease, including 282 cases (77%) of mitral valve repair and 84 cases (23%) of mitral valve replacement. There were 442 cases of degenerative mitral valve disease, including 412 cases (93.2%) of mitral valve repair and 30 cases (6.8%) of mitral valve replacement. 793 cases underwent tricuspid repair and 482 cases underwent modified Maze procedure for atrial fibrillation.

### Short-term outcomes

There was no statistically significant difference among the three groups in mortality, stroke, cardiopulmonary bypass time, aortic cross clamp time, mitral valve repair convert to mitral valve replacement, number of aortic cross clamp ≥2 times, unplanned secondary surgery during hospitalization (including thoracotomy hemostasis, thoracotomy exploration, redo mitral valve surgery, and debridement), intra-aortic balloon pump (IABP), extracorporeal membrane oxygenation (ECMO), continuous renal replacement therapy (CRRT), mechanical ventilation time, and intensive care unit length of stay during the perioperative period (Table 2).

**Table 2:** In-hospital outcomes and long-term outcomes.

	Morning group (*n *= 231)	Afternoon group (*n *= 543)	Evening group (*n *= 173)	*P* value
In-hospital outcomes				
CPB time, min	101.0 (83.0, 126.0)	104.0 (86.0, 130.0)	101.0 (83.0, 126.0)	0.241
ACC time, min	71.0 (57.0, 90.0)	74.0 (60.0, 94.0)	71.0 (55.0, 91.5)	0.131
Number of ACC ≥2 times, n(%)	4 (1.7)	14 (2.6)	5 (2.9)	0.697
MVr convert to MVR, *n*(%)	5 (2.2)	13 (2.4)	1 (0.6)	0.234
Mechanical ventilation time, h	17.0 (13.0, 18.0)	17.0 (13.0, 18.5)	17.0 (13.0, 19.0)	0.507
ICU length of stay, h	20.0 (17.0, 22.0)	20.5 (17.0, 22.0)	20.5 (16.8, 22.8)	0.834
Death, *n*(%)	2 (0.9)	8 (1.5)	2 (1.2)	0.768
Stroke, *n*(%)	2 (0.9)	5 (0.9)	2 (1.2)	0.953
Unplanned reoperation, *n*(%)	5 (2.2)	21 (3.9)	6 (3.5)	0.486
IABP, *n*(%)	1 (0.4)	8 (1.5)	2 (1.2)	0.401
ECMO, *n*(%)	0 (0.0)	4 (0.7)	1 (0.6)	0.240
CRRT, *n*(%)	3 (1.3)	8 (1.5)	3 (1.7)	0.938
Follow-up duration, months	54 (36, 69)	52 (35, 68)	52 (35, 70)	0.385
Long-term outcomes				
Death, *n*(%)	13 (5.6%)	35 (6.4%)	10 (5.8)	0.890
Stroke, *n*(%)	7 (3%)	26 (4.8%)	6 (3.5%)	0.474
Reoperation, *n*(%)	5(2.2%)	15(2.8%)	5(2.6%)	0.871

ACC, aortic cross clamp; CPB, cardiopulmonary bypass; CRRT, continuous renal replacement therapy; ECMO, extracorporeal membrane oxygenation; IABP, intra-aortic balloon pump; ICU, intensive care unit; MVr, mitral valve repair; MVR, mitral valve replacement.

### Long-term outcomes

**Figure 1:**
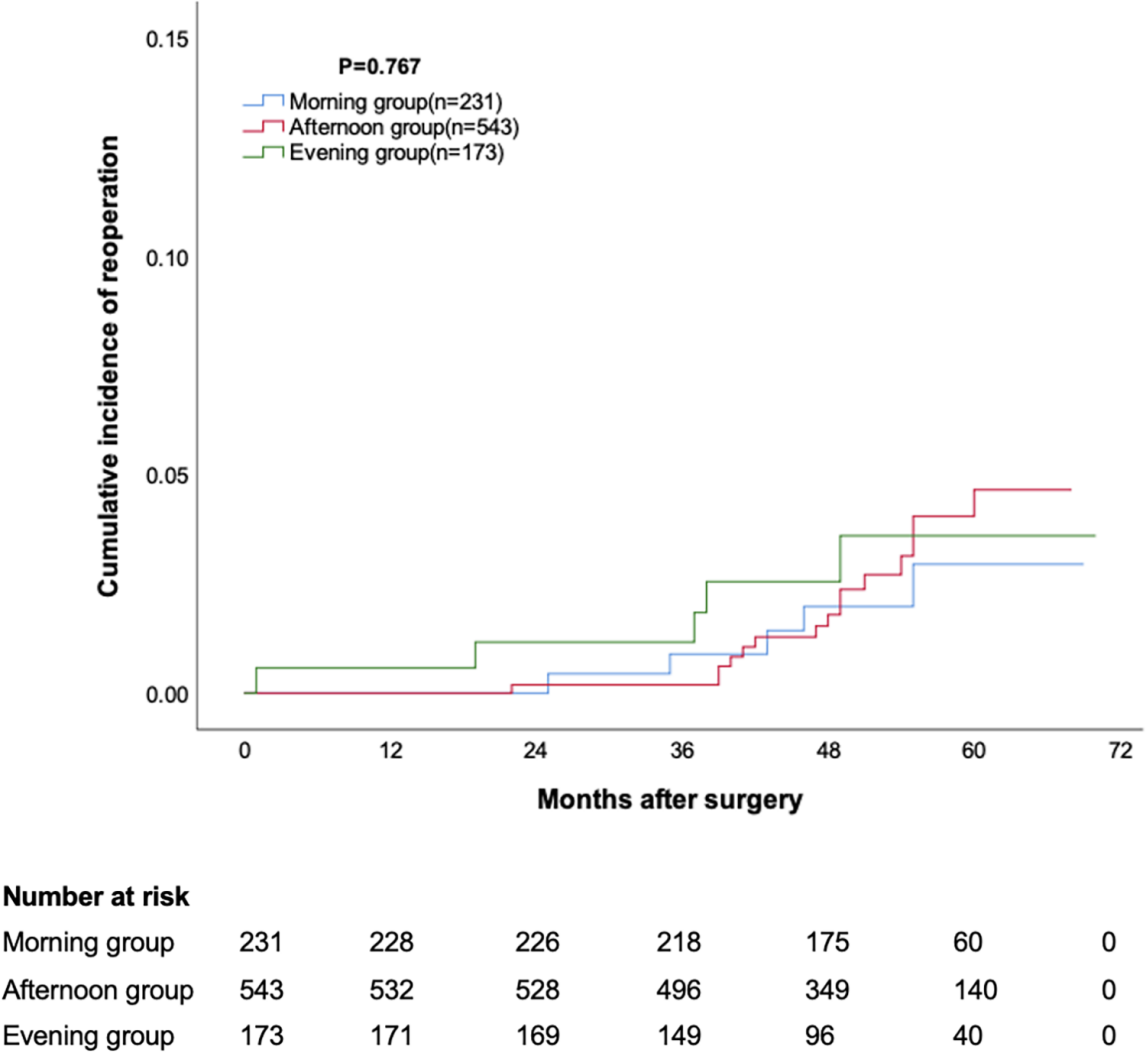
Cumulative survival in patients undergoing mitral valve surgery.

**Figure 2:**
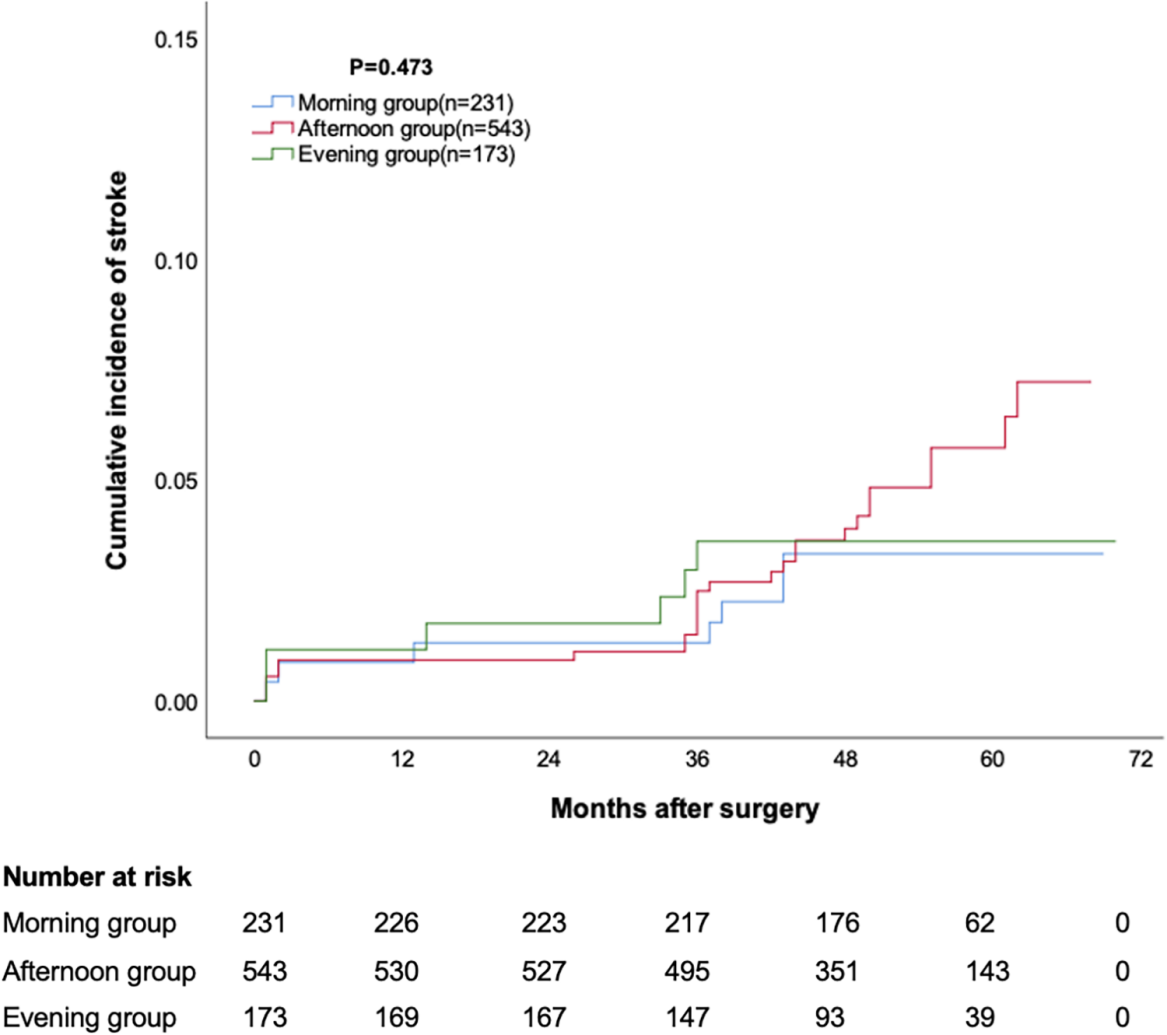
Cumulative incidence of stroke.

**Figure 3:**
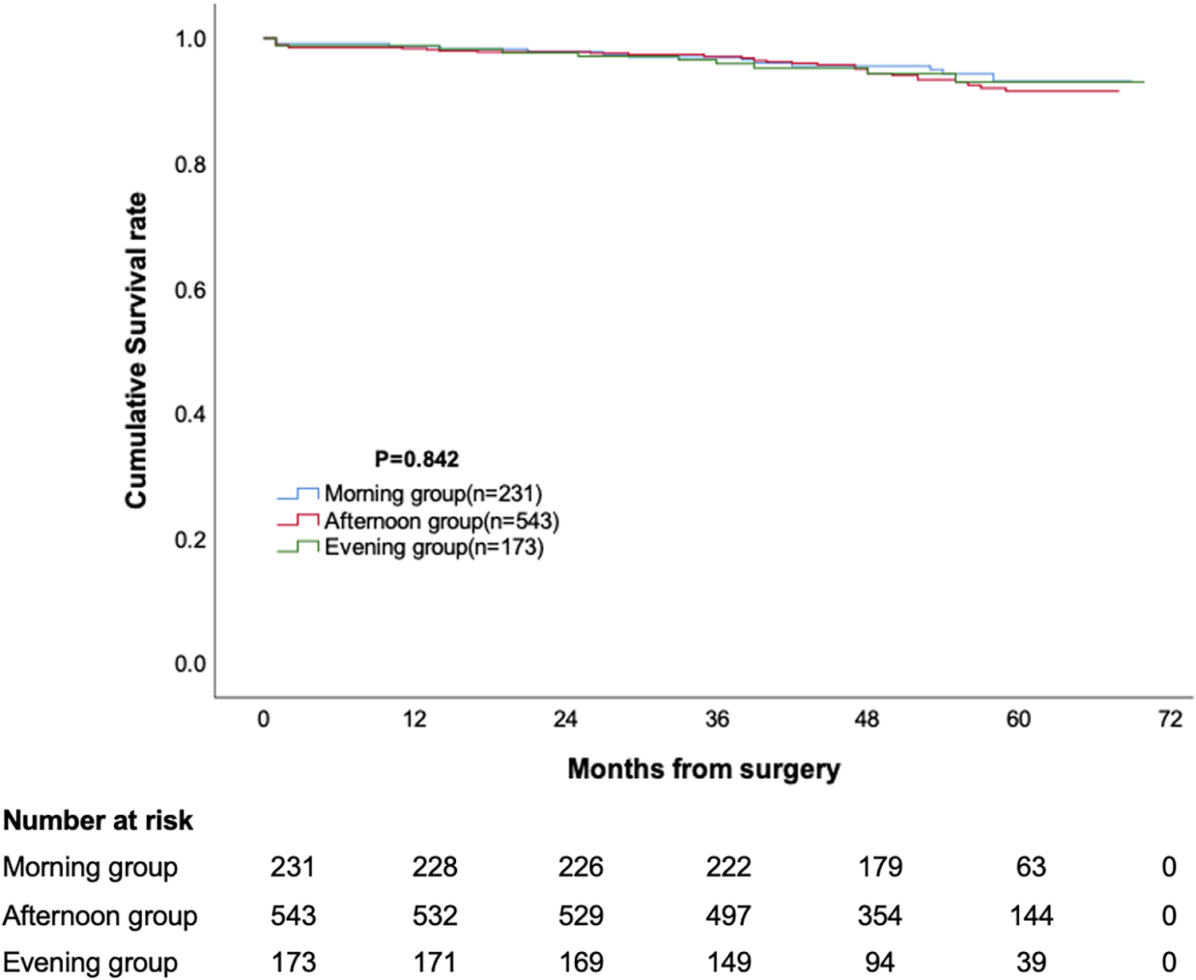
Cumulative incidence of reoperation.

The median follow-up duration for the three groups was 54 months, 52 months, and 52 months, respectively, with no statistically significant difference. The cumulative survival rate at 12, 24, 36, 48 and 60 months was 98.7%, 97.8%, 97.0%, 95.6% and 93.1% for morning group, 98.3%, 97.8%, 97.0%, 94.3% and 91.5% for afternoon group, 98.8%, 97.7%, 95.9%, 94.3% and 93.0% for evening group, respectively (*p* = 0.473, Figure 1). The cumulative incidence of stroke at 12, 24, 36, 48 and 60 months was 0.9%, 1.3%, 1.3%, 3.3% and 3.3% for morning group, 0.9%, 0.9%, 2.5%, 3.8% and 5.6% for afternoon group, 1.2%, 1.7%, 3.5%, 3.5% and 3.5% for evening group, respectively (*p* = 0.473, Figure 2). The cumulative incidence of reoperation at 12, 24, 36, 48 and 60 months was 0%, 0%, 0.9%, 2% and 2.9% for morning group, 0%, 0.2%, 0.2%, 1.8% and 4.5% for afternoon group, 0.6%, 1.2%, 1.2%, 2.5% and 3.5% for evening group, respectively (*p* = 0.767, Figure 3).

## Discussion

The present study provided the following findings. First, there was no significant difference in perioperative mortality rates and adverse events among patients undergoing mitral valve surgery in the morning, afternoon, and evening. Secondly, there was no significant difference in risk of long-term mortality, stroke and reoperation in patients undergoing mitral valve surgery in the morning, afternoon, and evening.

Since 2012, the number of cardiac surgery in Chinese Mainland has exceeded 200,000. By 2021, the number of cardiac surgery in Chinese Mainland has reached 278,000. The number of surgery in multiple hospitals has exceeded 3,000, and the number of cardiac surgery in Anzhen hospital and Fuwai hospital has exceeded 14,000, which inevitably leads to performing cardiac surgery in the evening frequently. The safety of evening surgery has become a concern and frequently questioned by many patient families. However, doctors often provide empirical answers to this question without reliable evidence-based medical evidence.

For the family members of patients, the question of whether the surgery time may affect the prognosis arises mainly from two aspects. Firstly, if the surgery is performed at night, the patient will fast for a long time and their physical function will decrease. The encoding process of mammalian proteins is regulated by circadian rhythms (9), which not only regulate multiple systems such as endocrine metabolism and autonomic nervous system, but also regulate the function of myocardial cells (10). Previous studies have shown that circadian rhythm can affect the area of myocardial infarction, with the area of nighttime myocardial infarction significantly exceeding that of daytime myocardial infarction (11, 12). Moreover, for patients with myocardial infarction, compared to daytime intervention, nighttime coronary intervention therapy has a poor prognosis (13). In addition, whether it is non cardiac surgery (14) or cardiac surgery (15), patients undergoing nighttime surgery are more likely to experience cognitive impairment and sleep disorders after surgery. Based on these evidence, it seems that performing surgery during the day, especially in the morning, is more advantageous.

Secondly, as the saying goes “morning is the best time of the day”, the doctor's energy in the morning is the best. After a long morning of work, afternoon energy and evening energy may be weaker and doctors may become more fatigued. Fatigue and insufficient sleep can cause cognitive decline and emotional changes in doctors, affecting their own health (16). Extended-duration work and fatigue were associated with an increased risk of significant medical errors (17). However, the vast majority of studies currently believe that insufficient sleep or fatigue among surgeons does not increase the risk of postoperative death and complications for patients (16). Even if the surgeon is on night shift (18) or undergoes night surgery (19) the day before the surgery, there is no increase in the risk of patients undergoing surgery the next day.

Several studies have compared the clinical outcomes of cardiac surgery performed at different time periods. In a cohort study (2), 298 patients underwent aortic valve replacement (AVR) in the morning and 298 patients underwent AVR in the afternoon. The results showed that the perioperative levels of troponin I in patients undergoing afternoon surgery were significantly lower than those undergoing morning surgery. After an average follow-up of 500 days, patients who underwent afternoon surgery had a significantly lower risk of major cardiovascular adverse events (cardiac death, myocardial infarction, and readmission of acute heart failure) compared to those who underwent morning surgery. To reveal its mechanism, further cytological and animal studies have found that the circadian rhythm of gene expression enhances the ability of myocardial cells to resist ischemia-reperfusion injury in the afternoon. Martin Michaud et al. (4) from Canada reached different conclusions, with 538 patients undergoing AVR or AVR plus coronary artery bypass grafting being matched 1:1 into the morning surgery group and the afternoon surgery group. Their results showed that there was no significant difference in plasma troponin I levels between the two groups after surgery. There was no significant difference in the incidence of adverse events such as death, myocardial infarction, low cardiac output, and stroke between the two groups of patients 30 days after surgery (4). Two other studies with a larger sample size also found that circadian rhythms have little impact on the prognosis of cardiac surgery. Nemeth et al. (5) analyzed 14,078 patients with AVR or coronary artery bypass grafting in the STS database and divided them into the morning surgery group and the afternoon surgery group based on the time they entered the operating room. There was no significant difference in perioperative mortality, stroke, ventilator-assisted time, renal failure, incision infection, reoperation, myocardial injury, new onset atrial fibrillation, and readmission between the morning and afternoon group (5). Kenny et al. (3) in the Netherlands matched 4,924 patients with AVR and/or coronary artery bypass grafting according to morning and afternoon surgeries. There were no significant differences in the 30 day postoperative mortality rate, perioperative myocardial infarction incidence, new postoperative atrial fibrillation, long-term mortality rate, and myocardial enzyme levels between the morning and afternoon surgical groups (3). Several studies on other cardiac surgery also conducted similar conclusion. The mortality for nonemergent coronary artery bypass grafting is independent of the timing of surgery. And circadian variation does not influence the outcome in coronary artery bypass grafting patients (20).

Our study is consistent with most previous research findings that surgery at morning or afternoon had similar prognosis in patients with cardiac surgery. Unlike previous studies, our study not only included daytime surgery, but also observed the impact of nighttime surgery on prognosis Furthermore, we observed long-term clinical results. The reason why most other studies have not paid attention to the impact of evening surgery on prognosis may be related to their surgery volume and work habits. The proportion of repair for degenerative mitral valve disease in this group is about 93.2%, which is consistent with the results reported in most literature (21). Since 2010, our center has carried out rheumatic mitral valve reconstruction surgery. Combining with the pathological characteristics of rheumatic mitral valve disease in Chinese people (7), we have gradually improved and summarized the standard as the “four step method” (8). The proportion of rheumatic mitral valve repair has been increasing year by year, and has achieved good long-term prognosis (6). Therefore, the proportion of mitral valve repair in patients with rheumatic mitral valve disease in this group reached 77%. In the present study, 60-month survival rate was 91.5%–93.1%, 60-month risk of stroke and reoperation were 3.3%–5.6% and 2.9%–4.5%, which comparable with the results of previous studies (22–25).

### Limitations

This study has the following limitations. Firstly, this study is a retrospective study, which may result in some unavoidable biases. Secondly, mitral valve disease often coexists with tricuspid valve disease and atrial fibrillation.

In order to reduce confounding factors, this study only selected patients with isolated mitral valve surgery or mitral valve surgery combined tricuspid surgery and surgery for atrial fibrillation as the research object, so it is unclear whether surgery at different time affect the prognosis of patients with other cardiac surgery. Furthermore, patients were divided into three groups, morning, afternoon and evening group. The grouping criteria are not exactly the same as those in other studies. Finally, in real-world clinical practice, some surgeries may started between two time periods according to our criteria. These patients were not included in the present study.

## Conclusions

In summary, our retrospective study suggest that there is no significant difference in the risk of short-term and long-term survival and adverse events after mitral valve surgery at different time periods in the morning, afternoon, and evening. Mitral valve surgery at night is safe.

## Data Availability

The raw data supporting the conclusions of this article will be made available by the authors, without undue reservation.
